# Commitment, Dominance, and Mate Value: Power Bases in Long-Term Heterosexual Couples

**DOI:** 10.3390/ijerph18041914

**Published:** 2021-02-16

**Authors:** Jitka Lindová, Tereza Habešová, Kateřina Klapilová, Jan Havlíček

**Affiliations:** 1 Department of Psychology and Life Sciences, Faculty of Humanities, Charles University, 182 00 Prague, Czech Republic; tereza.habes@seznam.cz (T.H.); katerina.klapilova@seznam.cz (K.K.); 2 National Institute of Mental Health, 250 67 Klecany, Czech Republic; jhavlicek@natur.cuni.cz; 3 Department of Zoology, Faculty of Science, Charles University, 128 44 Prague, Czech Republic

**Keywords:** power bases, dominance, mate value, commitment, relationship power

## Abstract

We assessed the relative contribution of economic, personal, and affective power bases to perceived relationship power. Based on evolutionary studies, we predicted that personality dominance and mate value should represent alternative personal power bases. Our sample was comprised of 84 Czech heterosexual couples. We measured the economic power base using self-report scales assessing education, income and work status. Personal power bases were assessed using self-report measures of personality dominance (International Personality Item Pool Dominance and Assertiveness subscale from NEO Personality Inventory-Revised Extraversion scale), and partner-report measures of mate value (Trait-Specific Dependence Inventory, factors 2–6). The first factor of Trait-Specific Dependence Inventory, which measures agreeableness/commitment was used to assess the affective power base. Our results show that perceived relationship power is associated with a perception of partner’s high agreeableness/commitment. Moreover, women’s personality dominance and mate value are also linked with perceived relationship power, which supports our evolutionary prediction of dominance and mate value working as power bases for women. The stronger effect of women’s than men’s power bases may be due to gender differences in investment into relationships and/or due to transition to more equal relationships currently sought by women in the Czech Republic.

## 1. Introduction

Interpersonal power belongs to the basic psychological concepts that describe social behavior, including romantic relationships [1,2], whereby unequal distribution of power in couples is one of the most studied predictors of decreased relationship quality [3,4,5]. It is of high importance to understand how power balance in romantic couples is determined by various sources of partners’ power. We use the term “relationship power” to describe the potential to exert influence or control in a relationship in relation to a partner [6]. In this context, influence is not restricted to a particular domain or time: it is generalized [7]. Previous research tended to focus on power bases, that is, the sources of power in relationships [8], including romantic ones, but the issue is still far from resolved. Some power bases can have a relatively broad impact within several types of interpersonal relations, while others are specific to a particular type, such as romantic relationships. Power bases in romantic relationships can take different forms. In addition to economic resources, various studies have also identified affective (level of involvement, commitment, or dependence), personal (personality, skills, appearance), normative (the social norm about which gender should have more power in a relationship), or cognitive (the perception of power) power bases [9].

### 1.1. Economic Power Bases

Power in romantic relationships has traditionally been explained by the resource theory [10], which views possession of resources as the main source of power. Although any valuable property can become a resource, research working within this framework tended to focus on socioeconomic resources such as financial means, occupational prestige, or social status. In a romantic dyad, the partner with fewer resources was thus supposed to be dependent on the resource-richer partner for access to resources he/she needs and wants [11]. Several early studies indeed found that men with a higher occupational prestige, income, or education tend to have greater relationship power ([12,13], but see also [14]) but that does not necessarily apply to women (e.g., [12,15]). In fact, in industrialized countries, differences in relationship power between men and women do not seem to be directly linked to differences in resources provided by either partner [16,17]. For these reasons, the role of socioeconomic resources as a power base in romantic relationships has been increasingly questioned.

### 1.2. Personal Power Bases: Personality Dominance and Mate Value

Personality, skills, and appearance can likewise predispose individuals to achieving higher relationship power [9,18].

Recently, predispositions to and the process of achieving a higher rank and influence in social groups became the subject of a number of studies by evolutionary researchers. It has been proposed that individuals can attain a higher rank within a hierarchy in two distinct ways: by dominance or by prestige ([19], but see also [20]). The mechanism of influence in these two scenarios differs. In the dominance-based approach, individuals strive for a higher rank by applying coercion, intimidation, aggression, and manipulation using rewards and punishments. In the prestige-based approach, a higher-ranking individual is recognized as competent and maintains influence by eliciting feelings of admiration, respect, liking, and social learning in others [21]. The distinction between dominance-based and prestige-based social rank was developed to explain the dynamics of larger groups. We suggest that similar mechanisms operate in the special case of power hierarchy in romantic relationships: the dominance-based way remains basically the same but the latter way of achieving high rank works by increasing mate value rather than via prestige. When speaking about dominance in this context, we should bear in mind a few things about this term. In particular, evolutionary psychology and evolutionary behavioral sciences tend to use the term “dominance” to describe a consistent pattern of interactional outcomes in a group or dyad in favor of one individual which are accepted by other individuals or by a partner [22]. To clearly differentiate between “dominance” in this sense from general disbalance of influence in a couple—which we, following the tradition of interpersonal psychology, call “relationship power”—we call the former “personality dominance”.

An association between personality dominance and leadership and influence has been confirmed by many studies [19,23,24]. There are also indications of an association between personality dominance and influence in dyads. A study by Schmid et al. [25] shows that, when assigned the lower hierarchical position in a dyad, individuals who are high in personality dominance behave more dominantly than individuals who are low in personality dominance. Also, it has been observed that personality dominance is positively associated with the degree of influence in dyadic laboratory tasks [26]. Nevertheless, the link between personality dominance and relationship power has been studied rarely and remains unclear.

In the case of mating, one can expect that mate value functions analogically to prestige. In a cooperative social group, characteristics which help individuals earn prestige are those that prove their competence and willingness to share it. Such characteristics, in particular skills and certain prosocial traits, are valued by others because they provide direct benefits to potential cooperative partners [27]. Similarly, mate value is based on a comparison between an individual and other members of the same sex with respect to characteristics—such as physical attractiveness or caregiving—which are valued by potential mating partners because they provide benefits in terms of higher reproductive success of those who mate with such individuals [28]. These mate value characteristics can be of various nature, including physical dispositions linked to health and fecundity (e.g., physical attractiveness and youth) [29], status and nurturance (e.g., physical prowess), but also psychological traits and skills predisposing an individual to caregiving, emotional support, and the like (such as nurturance, love, agreeableness, and emotional stability) [30,31].

When assessing mate value, one can either measure the absolute value of potential mate’s characteristics on a “mating market” or assesses the relative, dyad-specific value of a mate’s characteristics. Because relationship power is established relative to partner, the latter option seems more suitable here. One scale that measures mate value in this way is the Trait-Specific Dependence Inventory (TSDI) [30], where relative partner-specific mate value is assessed based on partner-rating, whereby the rating partner compares qualities of his/her mate to other possible mates available to him/her. Association between mate value as measured by the TSDI and relationship power has not been investigated directly as yet. Nonetheless, Ellis et al. [30] base their concept directly on the interdependence theory [32], which states that high dependence in a relationship is associated with high perceived outcomes obtained in such relationship on level of comparison with alternatives and propose that individuals partnered with high mate-value mates are more dependent and more invested in relationship. It has been shown that the less dependent partner holds more power in relationship than the more dependent partner, tends to make more personal sacrifices [33], and is more accommodating towards a partner [34].

### 1.3. Affective Power Basis

Affective resources, such as affection, love, dependence, and commitment [9,18] are also frequently studied in association with relationship power (e.g., [7,35,36]).

Some authors note that affection felt towards a partner and especially commitment are factors which make a person more willing to remain in a relationship, despite alternative mating opportunities and possible dissatisfaction with the relationship [37]. This idea has been long known as the “principle of least interest” [38] and in the context of romantic relationships, it can be summed up as predicting that the partner who is more invested, committed, and dependent on the relationships has less power [39]. Especially, commitment is a relation-specific motive: its aim is to ensure long-term persistence of attachment to an object of dependence [40]. It leads to a psychological experience of dependence or allegiance, where the more dependent partner has less power because he/she tends to be more accommodating and willing to sacrifice more. This prediction, i.e., that the more committed partners have less power in a relationship, has been confirmed by several studies [39,41,42].

Safilios-Rothschild [18], on the other hand, noted that in a relationship, affective resources—including affection, love, and needing the other—can be exchanged for socioeconomic or other resources and consequently decrease partner’s tendency to leave the relationship and lower his/her relationship power. Similarly, Ellis et al. [30] see agreeableness/commitment as a trait that is valued in individuals by their partners who profit from such individuals’ willingness to share and cooperate with them, make sacrifices, and be faithful. As such, they consider agreeableness/commitment as an important constituent of mate value of an individual. As mentioned above, due to the benefits which one’s high mate value brings for one’s partner, in particular benefits that would be difficult to be obtained with another partner, individuals partnered with high mate-value mates are expected to become more dependent in their relationship [30] and hold less power [43].

This reasoning views the effect of affection on relationship power in a contrasting way to the principle of least interest. Taken together, existing research is ambivalent on whether being loving, affectionate, and committed is a valuable trait that functions as a power base or, alternatively, whether it actually decreases relationship power by motivating individuals who are affectionate and committed to stay in a relationship. The TSDI [30] conceptualizes agreeableness/commitment as a mate value trait that can increase the dependence of partner of an individual who possesses it. Nevertheless, if an individual’s agreeableness/commitment motivates him/her to stay in a relationship and accommodate as predicted by the principle of least interest, then it seems that agreeableness/commitment is—despite being valued by one’s partners—linked with relationship power in an opposite way to other mate value traits.

### 1.4. Current Study

Research on the contribution of specific power bases to relationship power is extensive but most studies focus on the effect of just one type of power base. The relative impact of different power bases on the establishment of relationship power distribution in couples where different power bases need not be concentrated in one partner but distributed between both partners is as yet unknown. It is also unclear whether the same power bases are crucial for men and women or whether there are some gender differences at play here. In this study, we assess the relative effects of economic, personal, and affective power bases using a sample of Czech long-term couples.

More specifically, we aim to study the effect of common economic power bases, such as education, work status, and income, of affective power basis, and of two personal power bases in romantic relationships. The two personal power bases, namely personality dominance and mate value, are based on the proposed analogy to dominance- versus prestige-based achievement of higher rank in a social group. Because previous research suggests that economic, personal and affective power bases can all possibly increase relationship power, our basic prediction is that they all have a positive impact on the relationship power of men and women. Alternatively, though, it is also possible that, in contrast to socioeconomic characteristics, personality dominance, and mate value, affective feelings toward a partner have a negative effect on relationship power because they motivate individuals to remain in a relationship and retain partners despite eventual need to compromise own goals.

## 2. Materials and Methods

### 2.1. Participants

Our sample comprised 84 Czech childless heterosexual couples (168 individuals). They were of Czech nationality, with the exception of four Slovak partners of Czech individuals. Partners’ age ranged between 20–39 years (mean = 26.3 years for women, SD = 3.6; 27.4 years for men, SD = 4.1). The average relationship length was 5 years and 4 months. Thirty nine percent of the participants had at the time of the study completed college/university. Participants were recruited via fliers at 25 Prague gynecologists’ offices to participate in a larger study Intimate Behavior in Cohabiting Couples by Havlicek et al. [44]. We conceptualized long-term romantic relationships as steady dating or marriage [45]. In accordance with past research (e.g., [46]), we set inclusion criteria for participants at relationship length of at least six months and cohabitation of at least three months and age 18 to 40 (i.e., early and middle adulthood). In the final sample, all participants were dating for at least a year, and cohabited for at least the past six months. Although university students formed part of the sample, we tried to acquire a heterogeneous group of participants and include couples from diverse socioeconomic backgrounds. Couples with children were not included because parenthood markedly affects some relationship power predictors in women, such as work status due to maternity leave or self-perceived attractiveness [47]. Minimum required sample size was calculated according to Ackerman and Kenny [48]. Based on previous studies, we estimated the expected size of actor and partner effects to beta >0.30. Alpha was set to standard 0.05 and desired power to 0.80. This resulted in a minimum required sample size of 76 couples. All participants were informed about the goals of our study and gave their informed consent. They received a remuneration of 2000 CZK (88 USD) for participation in the whole study. Ethical aspects of the study were approved by the Institutional Review Board of Charles University, Prague, Faculty of Science (No. 2009/7).

### 2.2. Procedure

Data for this study were collected as part of a three to four-hour long testing session which was part of the abovementioned larger study. The larger study primarily investigated the effects of the menstrual cycle on relationship satisfaction, mate retention, and sexual behavior, and included keeping diaries by women during several menstrual cycles, administration of several questionnaires to both partners and laboratory testing session for the couple. During the testing session, each partner separately completed several questionnaires including those used in this study and had an individual interview with an experimenter within which a question about relationship power was posed. Additionally, participants had a couple’s interview with the experimenter (K.K.).

### 2.3. Measurement

Participants completed a demographic and socioeconomic information form which inquired about their gender, age, length of relationship (in months), level of education (scale 1–8), work status (scale 1–6, ranging from employment with no subordinates to top managerial functions) and income (scale 1–9, including current salary range) [49].

#### 2.3.1. Perceived Relationship Power 

This was assessed using a single item, namely In your current romantic relationship, which one of you is more dominant/powerful? (in Czech, the question was: Kdo je ve vašem současném vztahu dominantní? We deliberately used the term “dominantní” [dominant], which in Czech refers broadly to general influence superiority, instead of the Czech equivalent of powerful, “mocný, mít moc”, which is not used in everyday speech in that sense. Also, in Czech, the question clearly refers to relationship superiority and cannot be understood as asking about who of the two partners is the more socially or personally dominant individual in general) answered during an interview which the experimenter conducted with each partner separately. A single-item measure of relationship power is frequently used in couples’ research. Its aim is to capture the generalized perception of power balance over all domains [39,50,51,52,53]. The question was a part of an interview where it was immediately followed by questions about the spheres of relationship power of either partner. It appeared in the second part of the interview, after several initial questions about participants’ feelings about questionnaires they completed at home and their satisfaction with the testing session so far, and before questions about relationship satisfaction, mate retention, and sexuality. The question was complemented by specification of the meaning of dominance/power: participants were told it refers to who has more say in relationship. Participants were always encouraged to specify whether they feel somewhat more/less or clearly more/less powerful relative to their partner. Answers were transcribed from a recording of the interview on a scale of 1–5 (1–low relationship power, 2—somewhat low relationship power, 3—equal relationship power, 4—somewhat high relationship power, 5—high relationship power) by an experimenter (T.H.). Answers regarding the spheres of relationship power or other topics other than the perception of relationship power were not analyzed for the purposes of this paper.

#### 2.3.2. Personality Dominance 

This was assessed by two measures. The first was the dominance scale from International Personality Item Pool [54] (http://ipip.ori.org/) (accessed on 1 December 2016), an 11-item scale based on Gough’s California Psychological Inventory narcissism scale [55] (translated to Czech by our team) with reliability of alpha = 0.82. The scale included items such as I am quick to correct others, I impose my will on others and was answered on a 7-point Likert scale (1—definitely no, 7—definitely yes). The range of possible scores to achieve in this questionnaire are 7 to 77. Additionally, we also applied the 8-item Assertiveness subscale of Extraversion (NEO-PI-R [56]; Czech version by Hřebíčková [57]) where items such as I am dominant, forceful, and assertive are answered on a 5-point Likert scale (0—strongly disagree, 1—disagree, 2—neutral, 3—agree, 4—strongly agree). For the Czech sample, the reliability of this scale was 0.80 [57].

#### 2.3.3. Mate Value and Agreeableness/Commitment 

These were measured using the Trait-Specific Dependence Inventory [30] (TSDI; translated to Czech by our team and previously used, e.g., by Kučerová et al. [58]). This measure assesses comparisons between present and alternative romantic partners along several theoretically derived dimensions of mate value, including commitment, namely Agreeable/Committed (using adjectives such as considerate, devoted; factor 1), Resource Accruing Potential (professionally successful, responsible; factor 2), Physical Prowess (strong, athletic; factor 3), Emotional Stability (relaxed, calm; factor 4), Surgency (bold, outgoing, assertive; factor 5), and Physical Attractiveness (factor 6). The measure asks If you and your current partner broke up, how difficult would it be to find another partner who is as (adjective)? In other words, it assesses partner’s characteristics which contribute to his/her mate value relative to alternatives available for the rater. Answers are given on a five-point scale (1—not difficult at all to 5—extremely difficult). A high score on a given mate value scale indicates that the participant is highly valued by partner with respect to this characteristic. Alpha coefficients for individual scales in men and women are between 0.63 and 0.91.

For reasons presented above, instead of including the Agreeable/Committed factor in this measure of mate value as its integral part, we used it separately from the rest of the inventory to measure the affective power basis.

### 2.4. Data Analysis

We performed an exploratory correlational analysis between the primary scales measuring economic power (Education, Income, and Work Status), dominance (IPIP Dominance and NEO-PI-R Assertiveness), and mate value (factors 2 to 6 from TSDI). Significantly correlated scales measuring the same power base were combined into composite scores.

To identify the effect of actor and partner predictors and covariates on the dependent variables, we applied the Actor–Partner Interdependence Model (APIM) for dyadic data [59]. The APIM provides separate but simultaneous estimates of the actor and partner effects of members in a dyad [60]. Within the dyads, we distinguished between men and women. As independent predictors we included a socio-economic power variable, an agreeableness/commitment scale which assesses affection to partner, two personality dominance scales and a mate value variable. These variables served as both actor and partner predictors, depending on whether one’s characteristic or one’s partner’s characteristic were supposed to predict the dependent variable: one’s relationship power. Age was included as a covariate. Mutual independence of predictors was assessed by a correlational analysis.

## 3. Results

Of the total of 84 couples, 48 (57%) agreed on couple power balance. Partners tended to slightly overestimate their power: 25% reported low or somewhat low relationship power, 38% reported equal power, and 37% reported high or somewhat high relationship power. Descriptive statistics for all primary scales are provided in Table 1, which also shows gender differences in these scales calculated using pairwise t-tests and Kendall correlations between partners. Age was highly correlated between partners, which is why in the model, we used the couples’ mean age as a covariate and not as a dyadic variable. Length of relationship was correlated with participants’ average age, and was, therefore, not considered in analyses.

Table 2 shows intercorrelations between the primary scales. Scales measuring economic power bases (Education, Income, Work Status) were significantly intercorrelated and therefore combined into a composite Economic Power score by averaging z-scores of the three primary scales. Intercorrelated mate-value scales were averaged to create a Partner-Reported Mate-Value score. The two measures of dominance, IPIP Dominance and NEO-PI-R Assertiveness, which were not mutually correlated, were entered into the APIM as separate predictors.

**Figure 1:**
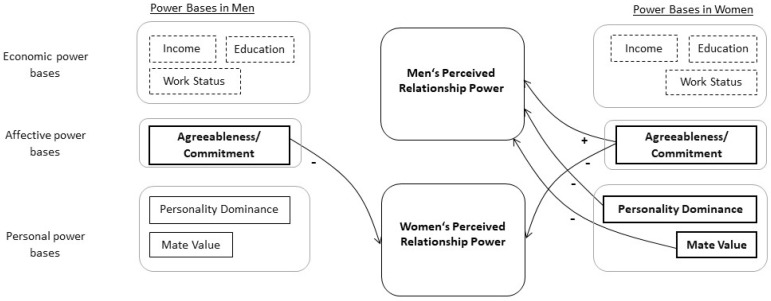
Relationship power bases: Result overview.

We performed the APIM to explore the effect of men’s and women’s Economic Power and affective (Partner-Reported Agreeableness/Commitment) and personal power bases (two scales of personality dominance: IPIP Dominance and NEO-PI-R Assertiveness and Partner-Reported Mate Value score) on their (actor) and their partner’s Perceived Relationship Power. We also included couple’s average Age as a covariate. The R^2^ of the APIM was 0.24 for men (with men’s Perceived Relationship Power as a dependent variable, men’s behaviour as actor variable, and women’s behaviour as a partner variable), and 0.31 for women (with women’s Perceived Relationship Power as a dependent variable, women’s behaviour as actor, and men’s behaviour as partner variables). Partial correlation for Perceived Relationship Power controlling for actor and partner variables and the covariate was statistically significant (r = −0.54, *p* < 0.001). Interaction between gender and actor effects was statistically significant (χ^2^ (5) = 13.0, *p* = 0.023), while interaction between gender and partner effects was not statistically significant (χ^2^ (5) = 7.2, *p* = 0.21). A combined test of interactions between gender and both actor and partner effects was statistically significant (χ^2^ (10) = 19.0, *p* = 0.040). Results for the individual predictors of the APIM are listed in Table 3 (see also Figure 1). 

Our results show that both men and women with higher Perceived Relationship Power viewed their partners as high in Agreeableness/Commitment. In addition, women with a higher Perceived Relationship Power were viewed by their partners as low in Agreeableness/Commitment. Moreover, women with higher personality dominance as measured by the IPIP Dominance score and with higher partner-rated Mate Value had male partners with a lower Perceived Relationship Power. Other proposed power bases had no significant effect on the Perceived Relationship Power.

To assess the robustness of our findings, we also performed a categorical regression model which included men’s and women’s power bases as predictors and within-dyad variable Relative Power as a dependent variable. The variable Relative Power was obtained by calculating the difference between men’s and women’s Perceived Relationship Power. The situation where both partners reported high relationship power relative to their partner was with respect to Relative Power the same as when both reported equal relationship power. In both cases, their score in Relative Power was zero. Higher scores indicated that the man had more power than the woman. The pattern of results was highly similar to the one obtained by the APIM and is reported in the Supplementary Materials (Table S1).

## 4. Discussion

Our analysis aimed at measuring the relative contribution of economic, affective, and personal power bases to the distribution of relationship power between partners in romantic couples. Based on the results of research in evolutionary psychology which investigated strategies used to acquire a high position in a hierarchy, we hypothesized that personality dominance and mate value could be important personal power bases. We found that, generally speaking, variables measuring affective and personal power bases are stronger predictors of perceived relationship power than variables related to economic power bases were. Specifically, the perception of one’s partner’s higher agreeableness/commitment predicted one’s higher perceived relationship power. Additionally, women’s self-reported personality dominance and mate value as perceived by their male partners was associated with their partner’s lower perceived relationship power. Economic status did not have a significant effect on power perception in the couple.

### 4.1. Economic Power Bases

Economic power bases, namely work status, income, and education, did not importantly contribute to perceived relationship power in our sample. This is in accord with recent criticism of the view that economic resources are a key constitutive factor of relationship power. This criticism came from gender scholars who note that even where women achieve higher income or status than their male partners, it often does not reverse the power dynamics in their relationships [15,16,17]. According to Bittman et al. [61], this may be because women tend to compensate for deviating from the gender norm by investing more in their relationships. This could be the case also in our sample, where women who have a relatively high economic status—but do not score high in personality dominance—may try to pursue the more traditional, low power position in a relationship to counterbalance the perceived deviation from the norm [15]. We did not include a measure of normative power bases, that is, a questionnaire measuring attitudes towards the traditional gender biased model of relationship power (see [62] for a review), to explore this possibility further, but future research should address this question.

Another possible interpretation is that in our relatively young sample, some participants have not yet finished their education and did not start building their careers. It is possible that this led to a mismatch between the effect of participants’ mate value, which is partly based on future status potential, and the effect of status they had achieved so far. Moreover, it may be that economic status plays a stronger role in couples who have children because their expenses and the difference in earnings between partners increase. It should also be noted that in our sample, status variables were relatively closely correlated between partners, and low variability in data which characterize differences between the partners’ scores may have decreased a chance to detect a possible effect of status on relationship power.

### 4.2. Personal Power Bases

Both assessed personal power bases, namely personality dominance and mate value, were associated with increased women’s relationship power relative to a partner. Our results are thus in agreement with predictions which distinguish between a dominance-based and prestige-based way of attaining a higher social rank in a group [19]. In romantic relationships, personality dominance may play a similar role of helping an individual acquire higher position directly by assertiveness or even coercion as in any other social group. It would be an effective but risky strategy, because partners might want to respond with a negative confrontation or even end any association with such an individual. The effect of mate value, on the other hand, could be viewed as a special case of the prestige-based way of achieving a high position in a group. In contrast to dominance, this strategy does not seem risky because mate value, like prestige, is supposed to be associated with partner’s freely conferred deference [21,30].

Associations of personality dominance and mate value with relationship power were, however, significant only in the case of partner effect of women’s behavior on men’s perceived relationship power. This is surprising not only because we are unaware of any published scientific explanation of why women’s—as opposed to men’s—characteristics should play a more important role in the establishment of relationship hierarchy but also because personality dominance is a trait often reported as more preferred and more valued in men than in women [63]. One would have thus rather expected personality dominance to function as a stronger power base with respect to relationship power in men than in women.

One possible interpretation of this finding could be that women with higher personality dominance direct their efforts at influencing the relationship more than men do because women generally invest more effort and energy into relationships than men do [64] (see also below). This is supported by studies which confirm that relationship success is more central to women’s than men’s self-concept [65], that women spend more time performing activities connected to family [66], and that women talk about family and relationship topics more often than men do [67]. It would then follow that personal characteristics such as dominance and mate value might serve as a basis of woman’s dominant behavior in a dyad, whereby the male partner would perceive a congruently lower relationship power.

Another possible explanation is based on the traditional and still prevalent social norm in Czech society where men tend to rank higher in the social hierarchy, although in recent times, the situation has been changing. Women are gaining more power both in society in general and in family in particular [68]. It may be that it is mainly up to the women and determined by the level of their individual personality dominance and/or mate value whether they comply with the traditional social norm of men’s higher position and are willing to accept lower power or pursue a different power arrangement. Future research including normative power bases among assessed potential sources of relationship power could decide between these interpretations.

Our results are in line with an independent research based on a similar sample [46] which indicates that women in Czech couples tend to have relatively high relationship power. That study also showed that male (but not female) control over their female partners and male personality dominance is associated with couple’s lower relationship quality. Numerous previous studies linked male dominance to coercive behaviors, including aggression, which has a negative impact on relationship satisfaction as perceived by female partners [69,70]. Taken together, it seems that while personality dominance in women serves as a power base that helps increase their relative power without decreasing perceived relationship quality by either partner, in men, personality dominance decreases relationship quality without having much effect on the distribution of power between the partners. Note, however, that in our study, we did not focus on couples with extreme forms of coercive dominance, such as is more common in men. This might be another reason why the effect of male personality dominance on relationship power balance was relatively weak.

### 4.3. Affective Power Bases

For both genders, we found that one’s partner’s agreeableness/commitment was associated with one’s own higher perceived relationship power. Our results thus indicate that affective characteristics may play an important role when establishing or maintaining relationship power within a couple. Considering the direction of the association, our results are in line with the prediction of the principle of least interest, originally proposed by Waller [38] and later developed by other authors [38,40,43]. It predicts lower power in the more affectively engaged and committed partners. An alternative scenario predicted that agreeableness/commitment is positively associated with relationship power because it is a constituent of one’s mate value and as such can benefit one’s partner (e.g., by being more cooperative or faithful), making him/her more dependent on a person who provides these benefits [30]. We used the Agreeableness/Commitment factor of the TSDI identified by Ellis et al. [30] which is conceptualized as a mate value constituent to measure the affective power base. Nevertheless, our results indicate that in our sample, higher scores in TSDI Agreeableness/Commitment factor probably reflect an affective motivational state in participants which is then linked to lower relationship power. At the same time, agreeableness/commitment as a personality trait can be valued in a partner. Indeed, it seems to be well established that Agreeableness/Commitment is an important constituent of mate value [30]. It is therefore likely that not all mate value traits help their bearers increase their relative relationship power. In the case of affection and commitment, individuals who have those characteristics might even be valued because they are expected to occupy a lower power position, thus indirectly aiding the partner to achieve his/her goals. Alternatively, as noted by Safilios-Rothschild [18], affection might only function as a power base in individuals who themselves are less in love or less committed, because they tend to exchange their love and commitment for other resources.

We should also note that the negative association between agreeableness/commitment and relationship power is not necessarily due to a causal relationship where, for instance, relatively higher affective engagement makes an individual dependent on the partner and puts the more agreeable/committed member of the couple in a lower power position. It could also be that high relationship power decreases an individual’s commitment via, for instance, decreased relationship satisfaction or increased quality of mating alternatives, which is what Lennon et al. [35] found in their study. On the basis of a cross-sectional study such as the present one, we cannot draw any conclusions regarding the causality of associations we observed.

### 4.4. Limitations

This study has certain limitations. First of all, partners agreed on power balance in their relationship only in slightly more than one half of all couples. This level of agreement is identical to one that our team obtained based on a larger sample and a similar single question answered in the form of a questionnaire item [46]. The level of agreement we found was, nonetheless, higher than that obtained using a similar scale by Harvey et al. [71] (49%). Such disagreement could be due to the fact that power can change depending on the situation and domain of interest. It can be that individuals tend to hold more power in situations and domains they consider especially important and that leads to a moderate overestimate of own power relative to partner, as observed in our sample. We also performed an analysis (reported in Table S1 of the Supplementary Materials,) where the difference between partners’ perception of relative power is used as a dependent variable. This should partly control for partners’ disagreement in perceived power balance. Importantly, this analysis produced the same pattern of results.

Secondly, we used a cross-sectional study to test an assumption which implies a causal relationship. Consequently, other interpretations—in addition to those which view affective and personal variables associated with relationship power as power base that influence the power balance in a relationship—are possible. A longitudinal study, which would establish the effect of economic, personal, and affective predictors on a change of relationship power, would, however, be rather difficult to perform.

Methods based on self-report and convenience sampling also carry some limitations in terms of the generalizations potential of some of the results. The sampling procedure should not, however, produce any false positive results regarding associations of predictors with the dependent variable. Moreover, predictors and dependent variables are reported by both partners, which make it unlikely that the associations we obtained are merely byproducts of some individuals’ cognitive bias, such as for instance a tendency to present oneself in a positive light.

Finally, our sample included individuals in both early and middle adulthoods. It is possible that, when separated, these two age cohorts would show slightly different patterns of results. Although our study did not enable us to test this assumption because analyses performed on split samples would not have sufficient statistical power, future studies would benefit from looking into this issue.

## 5. Conclusions

This study investigates predictions based on several theories from interpersonal psychology and evolutionary psychology in an attempt to identify power bases which influence power balance in romantic couples.

We found that one’s perceived relationship power is positively associated with one’s partner’s agreeableness/commitment in both genders and negatively with one’s own agreeableness/commitment in women, and that men’s perceived relationship power is negatively associated with women’s higher personality dominance and mate value. Our prediction that two important ways of attaining relationship power are personality dominance and mate value was confirmed for women. Stronger associations found in women than men could be due to the fact that women tend to put more effort in their relationships than men do, but it is also possible that this finding reflects a societal transition to more equal relationships which are sought by women with dominant personality and/or high mate value. The strongest association we found, namely that between participants’ high relationship power and their partner’s agreeableness/commitment, is in line with the principle of least interest [38]. It seems that in romantic relationships, dominance hierarchy is most strongly determined by motivation to maintain the bond and the effects of individual personality and physical appearance are relatively weaker. Our results could contribute to a better understanding of the dynamics and causes of malfunctioning in romantic relationships.

## Figures and Tables

**Table 1:** Descriptive statistics of variables considered for APIM.

Variable	Men *n* = 84		Women *n* = 84		Paired T-Test	Kendall Correlation
	Mean	SD	Mean	SD		
Perceived Relationship Power	3.3	1.1	3.2	1.3	0.36	−0.59 ***
Age	27.4	4.1	26.3	3.6	3.14 **	0.65 ***
Education	4.9	1.1	4.9	0.9	0.10	0.31 **
Work Status	4.4	1.0	4.2	1.1	2.03 *	0.30 **
Income	5.3	2.2	4.2	2.1	4.87 ***	0.50 ***
IPIP ^1^ Dominance	45.7	10.4	43.9	8.3	1.26	0.05
NEO-PI-R ^2^ Assertiveness	17.1	4.2	16.4	4.7	1.01	−0.09
MV ^3^ Agreeableness/Commitment	3.8	0.8	3.6	0.6	2.18 *	0.09
MV Resource Accruing Potential	3.3	0.6	3.3	0.5	−0.10	0.13
MV Physical Prowess	3.0	0.7	2.8	0.8	2.32 *	0.07
MV Emotional Stability	3.7	0.7	3.3	0.7	3.91 ***	−0.12
MV Surgency	3.1	0.6	3.1	0.5	0.53	−0.03
MV Physical Attractiveness	3.3	00.9	3.2	0.8	0.53	−0.13

^1^ International Personality Item Pool. ^2^ NEO Personality Inventory-Revised. ^3^ Mate Value. * < 0.05. ** < 0.01. *** < 0.001. For details on scales, see the Methods section.

**Table 2:** Intercorrelations among primary scales assessing economic power bases, mate value, and dominance.

*n* = 168	Income	Work Status	TSDI3	TSDI4	TSDI5	TSDI6	NEO Assertiveness
Education	0.321 **	0.244 **					
Income		0.529 **					
TSDI ^1^ 2			0.163 **	0.298 **	0.382 **	0.203 **	
TSDI3				0.126 *	0.184 **	0.161 **	
TSDI4					0.311 **	0.167 **	
TSDI5						0.259 **	
IPIP ^2^ Dominance							0.15

^1^ Trait-Stait Dependence Inventory. ^2^ International Personality Item Pool. * < 0.05. ** < 0.01. Entries are Kendall correlations.

**Table 3:** Results of the Actor-Partner Interdependence Model establishing the effect of actor and partner variables and the covariate on Perceived Relationship Power.

	Men’s Perceived Relationship Power		Women’s Perceived Relationship Power		M ^3^ + W ^4^ Actor’s Perceived Relationship Power	M + W Partner’s Perceived Relationship Power
R ^2^	0.24		0.31			
	Actor Effect (M to M)	Partner Effect (W to M)	Actor Effect (W to W)	Partner Effect (M to W)	Actor Effect	Partner Effect
Economic Power	0.18	<0.01	−0.08	−0.03	0.04	−0.02
IPIP ^1^ Dominance	<0.01	−0.34 **	0.14	−0.20	0.08	−0.280 ***
NEO-PI-R ^2^ Assertiveness	−0.13	−0.01	0.21 *	0.06	0.04	0.02
Partner-Reported Commitment/Agreeableness	−0.14	0.52 ***	−0.43 ***	0.47 ***	−0.30 **	0.50 ***
Partner-Reported Mate Value	−0.05	−0.36 **	0.15	−0.11	0.07	−0.26 **
Age		<0.01		<0.01		<0.01

^1^ International Personality Item Pool. ^2^ NEO Personality Inventory-Revised. ^3^ Men. ^4^ Women. * < 0.05. ** < 0.01. *** < 0.001. Entries for the individual predictors are standardized betas obtained by generalized least squares analysis with correlated errors and restricted maximum likelihood estimation.

## Data Availability

The data presented in this study are available in the supplementary material.

## Supplementary Materials

The following are available online at https://www.mdpi.com/1660-4601/18/4/1914/s1, Table S1: Results of a categorical regression establishing the effect of men’s and women’s economic, personal, and affective power bases and a control variable on Relative Power of the man over the woman.
