# Supplementary material for: Commitment, Dominance, and Mate Value: Power Bases in Long-Term Heterosexual Couples
Source: Int J Environ Res Public Health. 2021 Feb 16;18(4):1914. doi: 10.3390/ijerph18041914 (PMC7920442; doi:10.3390/ijerph18041914)
Supplement: Supplementary file 1 [file ijerph-18-01914-s001.zip › Supplement/Table S1-ijerph.docx]

**Table S1.** Results of a categorical regression establishing the effect of men’s and women’s economic, personal, and affective power bases and a control variable on *Relative Power* of the man over the woman.

| R^2^ | | .31 | |  | |
| --- | --- | --- | --- | --- | --- |
|  | | **Men’s predictors** | | **Women’s predictors** | |
| Economic Power | | .13 | | .09 | |
| IPIP^1^ Dominance | | .12 | | -.28^**^ | |
| NEO-PI-R^2^ Assertiveness | | -.08 | | -.11 | |
| Partner-Reported Commitment/ Agreeableness | | -.28^*^ | | .54^***^ | |
| Partner-Reported Mate Value | | -.04 | | -.32^**^ | |
| Age |  | | <.01 | |  |

^1^ International Personality Item Pool. ^2^ NEO Personality Inventory-Revised. ^*^ < 0.05. ^**^ < 0.01. ^***^ < 0.001. Entries for the individual predictors are standardized betas.
